# Supplementary figures and images for: Glucose and Oxygen Levels Modulate the Pore-Forming Effects of Cholesterol-Dependent Cytolysin Pneumolysin from Streptococcus pneumoniae
Source: Toxins (Basel). 2024 May 21;16(6):232. doi: 10.3390/toxins16060232 (PMC11209487; doi:10.3390/toxins16060232)

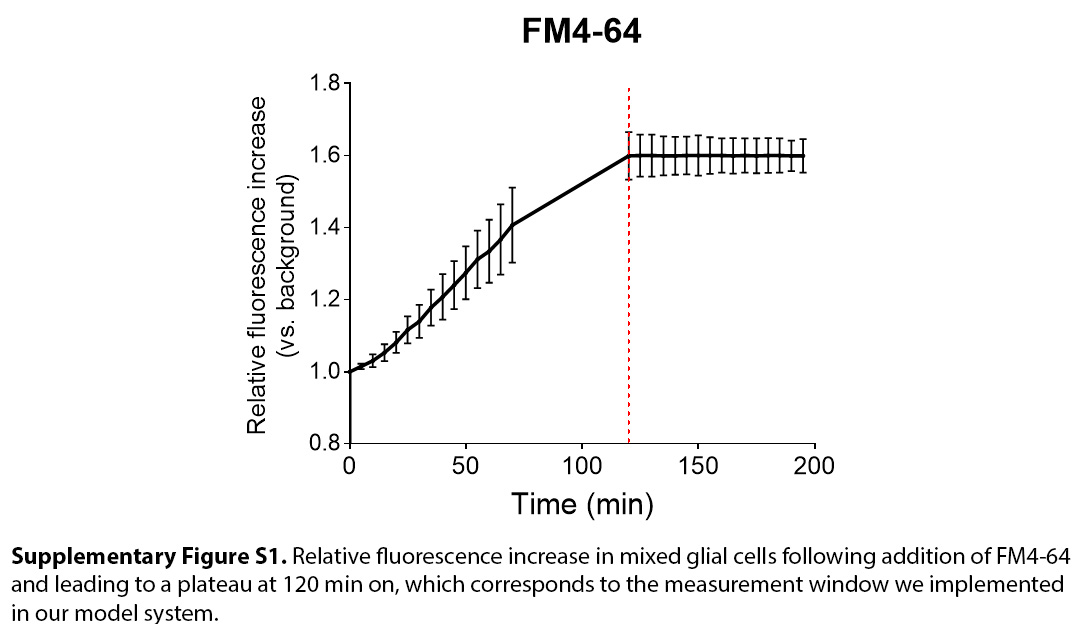

Supplement: Supplementary file 1 [file toxins-16-00232-s001.zip › Supplementary Figure S1.jpg]
